# Case of Hepatic Abscess, Involving the Gall Bladders, Successfully Treated by Injections of Solutions of Iodine

**Published:** 1871-07

**Authors:** J. A. Goldsberry

**Affiliations:** Annapolis, Indiana


ARTICLE VI.—*Case of Hepatic Abscess, involving the Gall Bladder, successfully treated, by injections of solutions of Iodine.* By J. A. GOLDSBERRY, M.D., Annapolis, Indiana.

June 16, 1866, I was called to see Hon. S. T. E., who had long been a sufferer from an abscess. He gave me the following brief history of his case:

On the 2d day of March, 1863, while at his place of business, (merchant) he was suddenly seized with an excruciating pain in the right side, just beneath the short ribs. The pain was deeply seated, and so severe as to render him, in a short time, almost helpless. There was soon noticed some enlargement of the liver, and in about two weeks from the day of the attack, there was observed a "*bulging*" in the iliac region of the right side. The pain was now of a gnawing or throbbing character, accompanied by violent rigors, alternating with flushes of heat, and some gastric irritation. After two months of terrible suffering, which had made sad havoc on a constitution naturally strong and powerful, there was discharged externally, a very large quantity of horribly fetid pus; this relieved materially, for the time being, the local symptoms, but so profuse was the daily discharge of matter, that for long weeks and months death seemed imminent almost hourly. In the meantime, pus burrowed among the abdominal muscles and sought new outlets. At the end of the sixth month, hydrothorax and a general dropsical condition supervened, which sequela, it was thought, rendered the case hopeless in the extreme. At the end of the first twelve months of his affliction, his *general* condition was thought to have improved a little; no amelioration, however, in the local trouble. Pus now passed out through three openings: one, the original outlet, situated about one inch to the right of, and three inches below, the umbilicus; one, a little below, and to the right of, this; and a third through the umbilicus itself. From this time up to the period when I was first called to see him, there was but little change in the local or constitutional condition.

Condition of patient on my first visit: Closely confined to his bed, and nearly helpless; very much emaciated; extreme debility; appetite capricious—sometimes voracious, at other times entirely wanting; bowels constipated; pus *occasionally* flowing through the umbilicus, and *constantly* from an opening situated just *above* Poupart's ligament, and about midway between the anterior superior spinous process and the spine of the pubes; the two other outlets spoken of having closed up sometime previous. Surrounding, and completely hiding from view the orifice of the lower sinus, was a large mass of fungous flesh, always highly sensitive and, at times, exceedingly painful. A sallow and jaundiced condition of the eye and skin; the features wan, worn and haggard, every lineament of the face expressive of the extremest physical suffering. Abdomen tympanitic, and the whole of its right side, from the upper margin of the hypochondrium down to the inguinal region, sensitive to the touch and painful on pressure. Condition of the kidneys apparently normal. He had just passed through a terrible paroxysm of pain, to which he was frequently subject, caused by the temporary closure of the orifice of the lower sinus. He informed me that, for three years and three months, he had been a sufferer; that through most of this period he had been under medical treatment; that his case had been examined by a number of medical gentlemen, and that, from time to time, he had had the benefit of superior medical advice; that life in his present condition was a burden; that though he had long since given up all hope of recovery, he thought it possible that something might be done at least to *mitigate* his sufferings, and smooth, to some extent, his pathway to the grave. He was willing, he said, to submit to any kind of treatment, however painful or unpleasant it might be, if there was a prospect of affording even *temporary* relief.

I learned that there had been a difference of opinion among the medical friends who had seen and treated the case, as to the real nature of the ailment. Some regarded it as a case of hepatic abscess, whilst others insisted that the disease originated in the lumbar vertebræ. After having carefully examined the case, and after having listened to my friend's very intelligent account of it, taking the following facts and symptoms connected with its history into consideration, I was led to the conclusion, that, whatever might be, or might have been, in the progress of the disease, its complications, and however serious in their nature, I had before me, primarily, at least, a case of abscess of the liver.

The patient was fifty-two years of age, of a sanguine-nervous temperament, and up to the present trouble, had all his life enjoyed remarkably good health. He was one of a large family, and all had been singularly free from diseases of a hereditary character; his parents were both living, healthy, active and vigorous. There was certainly present no hereditary taint, no tuberculous diathesis. The attack was *not* slow, gradual and insidious, as is common in spinal disease; no previous lameness; no soreness, nor even pain in the lumbar region; nor was the discharge, at any time, of that character which we find in diseases of a strumous nature; on the contrary, we find present several very valuable diagnostic signs in favor of hepatic abscess, viz: the manner of attack, being stricken down suddenly, in the midst of good health; the *locality*, intensity and character of the pain, enlargement of the liver, difficulty in breathing, and inability to lie on the left side. Of course my prognosis was as unfavorable as it could well be, and I frankly told my friend that his recovery was not at all probable, but that, in my opinion, something might be done to bring about, at least, *partial* relief, and thereby render his condition a little more comfortable.

Having informed him what would be my course of treatment, that it would necessarily be protracted and painful, and, after all, the result very uncertain, he promptly signified his willingness to submit to it, and insisted that I commence at once. Considering the fact that he had been subjected to a long course of constitutional remedies, indeed that the treatment had been almost *exclusively* constitutional, and being quite well satisfied that the disease was local in its nature; that it did not depend on any hereditary vice; that there was not now, nor had there been, any spinal trouble, no caries of the lumbar vertebræ or pelvic bones, but that the starting point was in the liver itself, and that in all probability there was still a serious lesion there; the intestinal lesion, for such there was, evidenced by the passage through one of the fistulous tracks of a very large ascaris lumbricoid; the hydrothorax and anasarca, which existed in the early history of the case; the frightful destruction wrought among the abdominal muscles, the consequent debility and prostration, were, all, so to speak, children of one common father, all drawing their nourishment from the same foul and contaminating source; hence I decided that my treatment should be addressed mainly to the local difficulty. The first step in the treatment consisted in removing from the orifice of the lower and principal sinus, the mass of morbid growth which seriously obstructed the free exit of matter, and which, for months, had been the source of indescribable suffering.

This I accomplished in the course of ten days by the daily application of argenti nitras. The orifice being fully exposed, I explored the sinus with the common silver pocket probe, carrying it upward and backward to the depth of four inches, when I encountered resistance of a soft, yielding nature, and found it utterly impossible, in all my subsequent explorations, to pass the probe beyond the point first reached. I was not induced, however, by this fact to even *hope* that I had arrived at the bottom of the canal, but was of the opinion, which the future history of the case painfully confirmed, that still farther upward and backward was to be found the real source of danger. I was now ready to begin the treatment proper, and though it extended through a period of many weary months, my therapeutics was exceedingly limited and simple.

For the first week I contented myself with injecting into the fistula, twice daily, tepid water, cleansing the track as thoroughly as possible. I substituted for this a weak solution of chloride of sodium, gradually increasing its strength, until more or less pain was produced. After having tested, as far as I could, the capacity of the sinus, and of the patient to *tolerate* the use of injections, I began the use of iodine, the remedy on which I expected to chiefly rely in my future treatment of the case, and in the use of which, I expected to succeed or fail in relieving my patient. The preparation employed was "Lugol's" solution, consisting of four grains of iodine to one pint of distilled water, and double the quantity of iodide potassium.

That I might now reach, if possible, with my injections, the source of the fistulous track, I prepared a small caoutchouc catheter; cutting it to the desired length, and clipping off the blind extremity, I passed it into the sinus to the distance of three and a half inches. I was not surprised to find that this procedure *increased* the *capacity* of the canal, that is, that I was thus enabled to throw the solution beyond the point previously reached. Prior to the introduction of the catheter, not quite two drams of fluid would fill the canal, *now* a half ounce was readily retained. The iodine injections were followed up once daily, for a period of six weeks, at the end of which time, the improvement in the case becoming so marked, I used it every second day.

About two months after taking charge of the case, I was not a little surprised, and, I might add, somewhat alarmed, on calling upon my patient, to find flowing from the fistula, what appeared to be, and which a close examination proved to be, *bile.* This was, to me, a new feature in the case, though my patient informed me that he had noticed a discharge of a similar character before, and that he had observed it, for the first time, three or four months before my connection with the case. Of course, there could not longer be any doubt as to the true source of the abscess. Doubtless the disease in the liver was extensive, and embraced that part of its under surface in relation with the upper surface of the body of the gall bladder.

During the long suppurative process in the liver, inflammatory action was set up in the wall of the gall bladder, resulting, by ulceration, in a perforation of its coats, hence, the presence of bile. It might be proper to say that the discharge of bile was not *constant*, but occurred only at intervals of every three or four weeks, and lasting each time from one to two days. I now increased the strength of the solution one-half, and continued to use it once every second or third day, up to the middle of November, the case having been in my charge about five months, at which period a small quantity of bile passed through the sinus *for the last time.*

The condition of the patient at this time was indeed encouraging; his general health had greatly improved, and the local trouble gave signs that were flattering and hopeful. He had dispensed with crutches, which he had been using for several weeks, and with the assistance of a cane, was now able to walk about the streets. The fistulous track was slowly but *constantly* contracting, evidenced by its diminished capacity and the necessity of substituting for the catheter used until now, one of less size, and the quantity of matter discharged was decreasing daily. The patient complaining but little from the injections, I now quadrupled the strength of the solution first used and employed it in this strength, once or twice every week, until, by reason of the gradually diminishing calibre of the fistula, it became impracticable to any longer introduce the syringe.

Sometime in the month of June, 1867, about one month after the last injection, and twelve months after treatment by their use commenced, all discharge ceased, and the fistula finally closed. A few weeks after this event, which I had labored and waited for with no little solicitude, and that my long-suffering patient so much desired to realize, he was suddenly attacked with a very severe pain, originating in the right hypochondrium, but soon locating itself in the stomach. The attack seemed to be of a neuralgic character, was attended with free emesis, and lasted from two to three hours. This was soon followed by another of like character, and for the next six months, at intervals of one, two or three weeks, these frightful and distressing paroxysms continued to come up, telling fearfully on his general health, and creating a good deal of anxiety in the minds of patient, friends and physician, as to the ultimate result. But with their cessation, the system promptly rallied, and soon regained all it had lost. Since that time the health of my friend has been excellent; his capacity for labor, both physical and mental, and his powers of endurance, are but little impaired. One, familiar with the severe and protracted struggle through which he has passed, to see him now, the very personification of good health and vitality, moving about with the freedom and facility of a sprightly boy, must be impressed with the fact that he is a striking example of the truth of that proverbial saying, " as long as there is life there is hope."

---

BOHEA-MIANS.—The feminines who tease for admission to " mixed clinics." In case of refusal, " *Gunpowder* " is threatened.

